# Case report of a pituitary thyrotropin-secreting macroadenoma with Hashimoto thyroiditis and infertility

**DOI:** 10.1097/MD.0000000000009546

**Published:** 2018-01-05

**Authors:** Jiaqi Li, Jianwei Li, Shu Jiang, Ruichao Yu, Yerong Yu

**Affiliations:** a Department of Endocrinology and Metabolism; b Department of Neurosurgery, West China Hospital, Sichuan University, Chengdu, Sichuan Province, China; c Department of Pathophysiology and Molecular Pharmacology, Joslin Diabetes Center, Harvard Medical School, Boston, Massachusetts.

**Keywords:** Hashimato thyroiditis, infertility, TSHoma, TSH-secreting adenoma

## Abstract

**Rationale:**

Thyrotropin-secreting adenoma (TSHoma) is rare, diagnosis and treatment are often delayed if the condition coexists with Hashimoto thyroiditis. The enlarged pituitary adenoma may eventually induce panhypopituitarism, infertility, or the compression of optic nerves and optic chiasma.

**Patient concerns:**

This patient was a 36-year-old man who had been referred to the pituitary disease multidisciplinary team (MDT) of the West China Hospital, due to infertility.

**Diagnoses:**

Examinations revealed pituitary thyrotropin-secreting macroadenoma.

**Interventions:**

We conducted trans-sphenoidal surgery. Human chorionic gonadotropin (HCG) and human menopausal gonadotropin (HMG) were used for reproductive reconstruction after surgery.

**Outcomes:**

This patient successfully fathered a child.

**Lessons:**

To date, the multidisciplinary team treatment of TSHoma was rare, TSHomas are often misdiagnosed as macroadenomas, because the clinical features are varied and it often takes a long time to be diagnosed. So the purpose of this case report is to attract attention to the manifestation of increased thyroid stimulating hormone (TSH) concentration and discuss MDT treatment for TSH-secreting adenoma.

## 1 Introduction

TSH-secreting pituitary adenomas (TSHomas) rarely cause hyperthyroidism.^[1,2]^ TSHoma, which accounts for 0.5% to 3% of all pituitary tumors,^[3,4]^ has been found in 1 case per million.^[5]^ Hashimoto thyroiditis is a common auto-immune endocrine disorder. The coexistence of Hashimoto thyroiditis with TSHoma may mask the full clinical features of thyrotoxicosis, making the diagnosis and treatment complicated. After a delayed period, the enlarged pituitary adenoma may induce panhypopituitarism or the compression of optic nerves and optic chiasma. This report describes the complicated case of TSH-secreting pituitary adenoma copresenting with Hashimoto thyroiditis and infertility while also detailing the management of these diseases by a multidisciplinary team.

## 2 Case report

**Figure 1:**
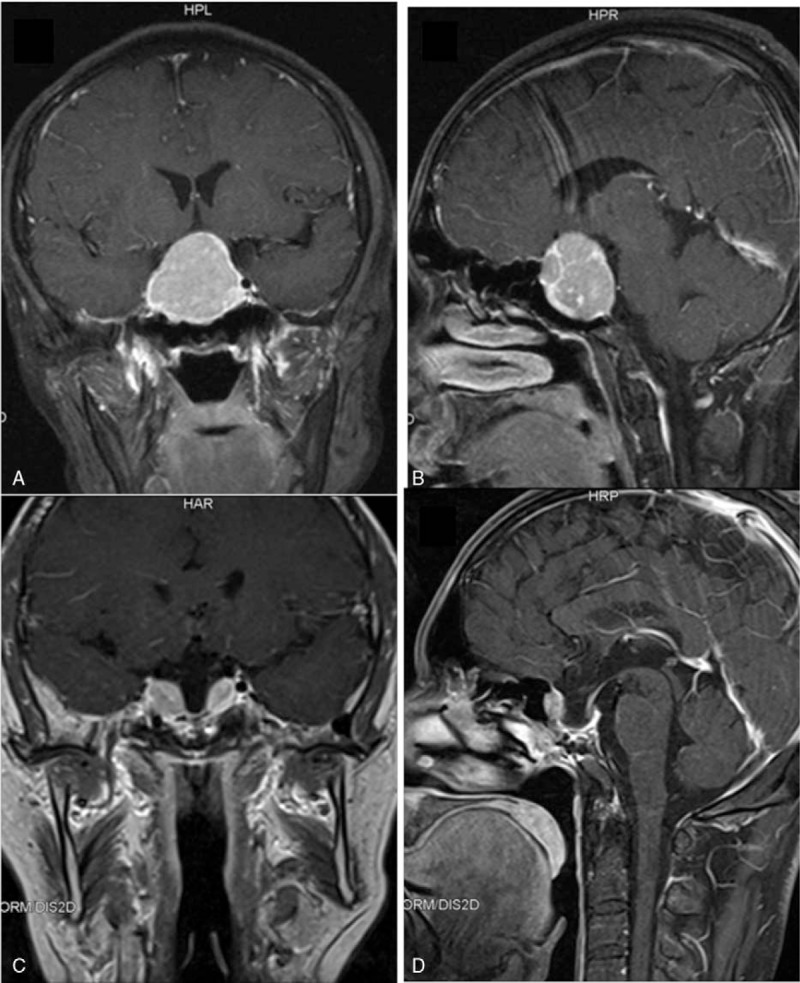
A pituitary tumor of 4.3 × 4.1 × 3.0 cm in the sella involving cavernous sinuses and extending inferior to the sphenoid sinus and third ventricle.

**Figure 2:**
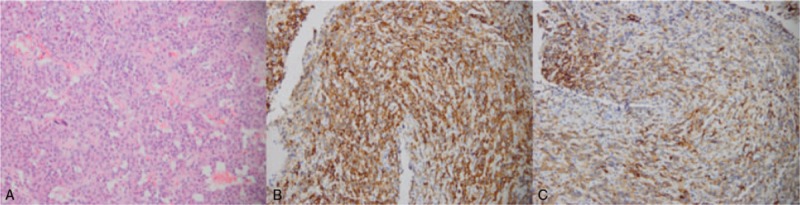
Histopathological findings: Hematoxylin and eosin staining for the cells in the surgical specimen showed diffuse proliferations of small atypical cells (A, ×200). Immunostaining for thyroid-stimulating hormone reveals the expression of TSHb (B, ×200), and the adenoma shows diffuse staining for antibody against prolactin (C, ×200).

A 36-year-old man was admitted to West China Hospital because of low libido, decreased morning erections, and loss of axillary and pubic hair that lasted 8 years. He had a history of binocular diplopia, increased stool frequency 7 years prior to admission, and weight loss of 10 kg. He also had thyroid nodules, and thyroid functional tests showed normal FT3 (7.41, normal 3.6–7.5 pmol/L), FT4 (21.56, normal 12–22 pmol/L), and increased TSH (24.79, normal 0.27–4.2 μIU/mL). He ignored the test result, because of the normal concentration of FT3 and FT4. Four years ago the patient got married and was referred to our hospital because of sterility. At this time, the hormonal examination implied central hypogonadism with serum levels of 1.6 mIU/mL FSH (RR, 1.5–12.4), LH 1.0 mIU/mL (RR, 1.7–8.6), E2 <5 pg/mL (RR, 7.63–42.8), and T <0.03 ng/mL (RR 2.49–8.3). Magnetic resonance imaging (MRI) of the sella region was therefore performed, revealing a pituitary tumor measuring 4.3 × 4.1 × 3.0 cm in the sella, involving cavernous sinuses and extending inferior to the sphenoid sinus and third ventricle (Fig. 1). He was referred to our multidisciplinary team of pituitary disease and transferred to department of neurosurgery. On physical examination at admission, the patient was 168.0 cm tall and weighed 59.0 kg with a body mass index (BMI) of 20.9 kg/m^2^. The patient's char-acteristics were as follows: temperature, 36.2 °C; pulse, 56 beat/min; blood pressure, 94/64 mmHg; respiratory rate, 20 breath/min. The patient presented with blurred vision. The TgAb and TPOAb were positive. The thyroid gland was diffusely enlarged and ultrasonography of the thyroid revealed a multinodular gland with a moderate parenchymal heterogeneity suggesting Hashimoto thyroiditis. The laboratory hormone examination showed hypopituitarism (Table 1). The computer perimetry suggested a defective visual field. Based on these data, we diagnosed the patient with TSH-producing pituitary macroadenoma, hypopituitary, and Hashimoto thyroiditis. Two weeks after diagnosis, total resection of the pituitary macroadenoma was performed through trans-sphenoidal neurosurgery in September 2014. Upon immunohistochemical examination, the resected pituitary adenoma cells exhibited positive staining with the TSH and PRL antibody, and the percentage of positive Ki-67 was <2%, suggesting that the tumor was benign (Fig. 2). After the operation, TSH and thyroid hormone rapidly decreased to a normal range. However, the concentration of ACTH, cortisol, LH, FSH, and testosterone were still extremely low. One year after the operation, this patient was attended to by our pituitary disease MDT team for reproductive reconstruction. We treated him with human chorionic gonadotropin (HCG) 2000 IU, qod and human menopausal gonadotropin (HMG) 75 IU, qod. Over the next 2 months, his sperm concentration increased, and finally his wife became pregnant naturally. The patient was administered hydrocortisone 20 mg/d, testosterone 80 mg/d, and followed up by our pituitary MDT team.

**Table 1:**
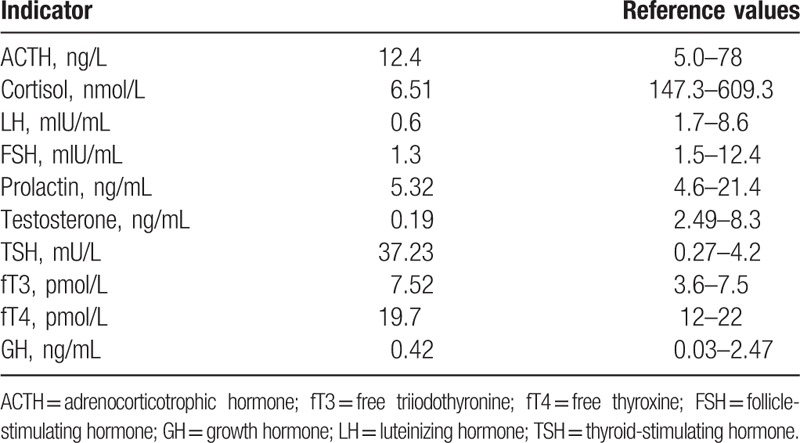
Hormonal test results.

## 3 Discussion

Herein, we report a patient who was diagnosed with infertility caused by pituitary TSH adenoma. To the best of our knowledge, no previous cases have been reported where reproductive reconstruction of TSH-producing adenoma was performed after surgery. Since 1960, a total of about 350 cases of the TSH-secreting adenoma have been described worldwide.^[6]^ The clinical manifestation of this disease occurs in disparate age groups. The pathology equally involves women and men.^[7]^ In most cases, TSH-secreting adenomas are characterized by secretion of TSH only.

As was the case with our patient, TSHomas are often reported as macroadenomas after a delayed period, because the clinical features are varied and it often takes a long time to be diagnosed.^[8,9]^ Typically, the TSHoma manifests with mild symptoms of thyrotoxicosis along with atrial fibrillation, heart failure, and transient paralysis. It is also associated with abnormal levels of thyroid hormones and TSH,^[10]^ and is considered a “syndrome of inappropriate secretion of TSH” (SITSH) or “central hyperthyroidism.” However, due to insufficient production of thyroid hormones possibly in association with auto-immune damage to thyrocytes, the coexistence of Hashimoto thyroiditis and TSHoma may dissimulate the full clinical manifestation of thyrotoxicosis, and result in the biochemical remission of hyperthyroidism. In the meantime, the negative feedback from hypothyroidism may result in an increase in the level of TSH and the size of the pituitary adenoma over years.^[11]^ The enlarged mass may cause visual field defects (40%), headaches (20%), and mild or severe loss of pituitary functions. Partial hypogonadism occurs in about 1/3 of patients; it is mainly identified through menstrual irregularities in females and through central hypogonadism, delayed sexual development, and decreased libido in males.^[6]^ In our case, the laboratory findings indicated increased TSH, free T3, free T4, hypopituitarism (pituitary-adrenal axis, pituitary-gonad axis, and growth hormone defects), and visual confirmation of an adenoma-like mass through MRI, which are consistent with a TSH-producing adenoma, though the typical clinical signs of hyperthyroidism were absent.

After trans-sphenoidal adenomectomy, unplanned hypopituitarism occurred in approximately 5% of the patients, while 50% recovered normal hormonal function. New hypopituitarism or worsened pituitary function occurs most commonly in patients with a mass >2.0 cm in size.^[12,13]^ In our case there was gonadal, adrenal, and thyroid failure following surgery for a pituitary macroadenoma.

It is complex to manage male infertility in cases of macroadenoma due to the difficulty of imitating the pulsatile secretion of GnRH.^[14]^ The 2 methods that are generally available for this situation are subcutaneous GnRH injection pumps or intramuscular injections of HCG and HMG as replacements for luteinizing hormone (LH) and follicle-stimulating hormone (FSH).^[14,15]^ The first method requires an intact pituitary gland and can only be applied in cases of hypothalamic diseases.^[14]^ The second method is more commonly used.^[15]^ A previous report mentioned successfully fathered a child case with treatment of HCG/HMG injection,^[16]^ the results are similar in our case.

Our case presents a comprehensive therapy for pituitary adenoma executed by our pituitary disease multidisciplinary team (MDT). Our MDT reviewed each suspected case, implemented a personal management plan, and followed each case. This case suggests that MDT made optimal patient-centered decisions thus improving the clinical outcomes of pituitary adenoma in their patients.

## Acknowledgments

The authors acknowledge Dr Ying Tang of the Department of Pathology at West China Hospital of Sichuan University for helping with the pathological examinations. They also acknowledge Dr Yi Wei of West China Hospital of Sichuan University for performing the MRI report.
